# Telomerase inhibitor imetelstat has preclinical activity across the spectrum of non-small cell lung cancer oncogenotypes in a telomere length dependent manner

**DOI:** 10.18632/oncotarget.9335

**Published:** 2016-05-12

**Authors:** Robin E. Frink, Michael Peyton, Joan H. Schiller, Adi F. Gazdar, Jerry W. Shay, John D. Minna

**Affiliations:** ^1^ Hamon Center for Therapeutic Oncology Research, University of Texas Southwestern Medical Center, Dallas, TX, USA; ^2^ Inova Schar Cancer Institute, Falls Church, VA, USA; ^3^ Department of Pathology, University of Texas Southwestern Medical Center, Dallas, TX, USA; ^4^ Simmons Cancer Center, University of Texas Southwestern Medical Center, Dallas, TX, USA; ^5^ Department of Cell Biology, University of Texas Southwestern Medical Center, Dallas, TX, USA; ^6^ Center for Excellence in Genomics Medicine Research, King Abdulaziz University, Jeddah, Saudi Arabia; ^7^ Department of Pharmacology, University of Texas Southwestern Medical Center, Dallas, TX, USA; ^8^ Department of Internal Medicine, University of Texas Southwestern Medical Center, Dallas, TX, USA

**Keywords:** imetelstat, telomerase, telomeres, lung cancer, telomerase inhibition

## Abstract

Telomerase was evaluated as a therapeutic oncotarget by studying the efficacy of the telomerase inhibitor imetelstat in non-small cell lung cancer (NSCLC) cell lines to determine the range of response phenotypes and identify potential biomarkers of response. A panel of 63 NSCLC cell lines was studied for telomere length and imetelstat efficacy in inhibiting colony formation and no correlation was found with patient characteristics, tumor histology, and oncogenotypes. While there was no overall correlation between imetelstat efficacy with initial telomere length (ranging from 1.5 to 20 kb), the quartile of NSCLC lines with the shortest telomeres was more sensitive than the quartile with the longest telomeres. Continuous long-term treatment with imetelstat resulted in sustained telomerase inhibition, progressive telomere shortening and eventual growth inhibition in a telomere-length dependent manner. Cessation of imetelstat therapy before growth inhibition was followed by telomere regrowth. Likewise, *in vivo* imetelstat treatment caused tumor xenograft growth inhibition in a telomere-length dependent manner. We conclude from these preclinical studies of telomerase as an oncotarget tested by imetelstat response that imetelstat has efficacy across the entire oncogenotype spectrum of NSCLC, continuous therapy is necessary to prevent telomere regrowth, and short telomeres appears to be the best treatment biomarker.

## INTRODUCTION

Lung cancer is the leading cause of cancer-related death in both men and women in the United States [1]. Non-small cell lung cancer (NSCLC) makes up 85% of all lung cancer cases and can be further categorized as adenocarcinoma, squamous cell carcinoma and large cell carcinoma. Standard “doublet” first line chemotherapies include a combination of paclitaxel or docetaxel, pemetrexed or gemcitabine, and carboplatin or cisplatin with tumor response rates of approximately 20-30%. The recent advent of targeted agents such as erlotinib and crizotinib has yielded dramatic short-term clinical responses in patients with specific oncogene mutations (e.g. mutant EGFR or EML4-ALK fusions) showing the benefit of developing molecular analyses of tumors to implement “precision medicine” for lung cancer [2-4]. However, such approaches target only small subsets of patients with specific mutations and do not give curative clinical responses. Thus, new targeted therapeutic agents are needed, particularly for targets that are relevant to a large majority of lung cancers.

In this regard, telomerase is expressed in 85-90% of all cancers including lung cancer and is responsible for the limitless replication potential of cancer [5, 6]. By contrast, telomerase is not expressed in most normal somatic cells making it an attractive, almost universal, target for cancer therapy. Telomeres, located at the ends of chromosomes, erode with every cell division due to the end replication problem, eventually leading to cellular senescence when telomeres become critically short [7]. When telomerase is active, telomere ends are re-elongated or stabilized allowing for continued cell divisions. The two functional components of telomerase are hTERT and hTR (hTERC). hTERT is a cellular reverse transcriptase that extends the ends of telomeres. hTR is a functional RNA that contains a telomere template component used by the reverse transcriptase to add the appropriate TTAGGG telomeric repeats to human telomeres during each replication cycle to counterbalance the losses due to the end replication problem [8-10]. Precancerous cells typically have very short telomeres and need to bypass senescence and crisis to continue cell proliferation, and therefore almost 90% of cancer cells have short telomeres relative to normal cells [11, 12]. A subset of precancerous cells must turn on telomerase (or some alternative telomere lengthening mechanism) to become capable of extended growth potential. In addition, cancer stem cells (cancer initiating cells) need telomerase for their continued function [13]. Thus, targeted inhibition of telomerase in cancer cells should lead to critically short telomeres and subsequent senescence or cell death in tumor cells while causing minimal side effects on non-cancerous cells and could also be combined with chemotherapy or targeted therapy [13].

Imetelstat (formerly GRN163L) is a telomerase inhibitor that binds the hTR RNA template component of telomerase preventing telomerase from elongating telomeres. Imetelstat is a synthetic lipid-conjugated 13-mer N3′P5′ thio-phosphoramidate [14] (Supplemental Figure S1). Previous preclinical studies have shown efficacy of imetelstat in breast, prostate, liver, brain, pancreatic and bladder cancers as well as multiple myeloma and lymphoma [15-23]. Imetelstat has also shown efficacy in the A549 lung cancer cell line, but no additional preclinical work has been done to demonstrate imetelstat effectiveness in other NSCLCs [24]. Imetelstat has been tested in 14 clinical trials including a completed Phase I in NSCLC which determined safety and maximum tolerated dose in combination with paclitaxel and carboplatin chemotherapy as well as a randomized Phase II which used imetelstat as maintenance therapy for NSCLC [25]. What is not known is the efficacy of telomerase as an oncotarget in terms of the range of response to imetelstat in NSCLC preclinical models, whether there are correlations of imetelstat response with clinical characteristics, oncogenotypes, response to standard chemotherapy and targeted therapy agents, and whether imetelstat growth inhibition is associated with any tumor molecular biomarkers to facilitate a “precision medicine” based approach in future clinical trials.

In the present study, we looked at telomere length and growth responses to imetelstat in a large panel of NSCLC cell lines representing many different oncogenotypes as well as a large spectrum of standard chemotherapy and targeted therapy response phenotypes. We conclude that: imetelstat has activity (critically shortens telomeres, inhibits clonogenicity, and induces senescence) across the oncogenotype and standard chemotherapy/targeted therapy spectrum of NSCLCs; imetelstat response phenotypes correlate with tumor telomere length and treatment duration; and surprisingly, we found no evidence of acquired resistance with long-term therapy. These results validate telomerase as a relevant target and suggest that selecting patients whose tumors have the shortest quartile of telomeres may serve as an enrollment biomarker for telomerase inhibitors.

## RESULTS

### NSCLCs exhibit heterogeneity of telomere length

**Figure 1:**
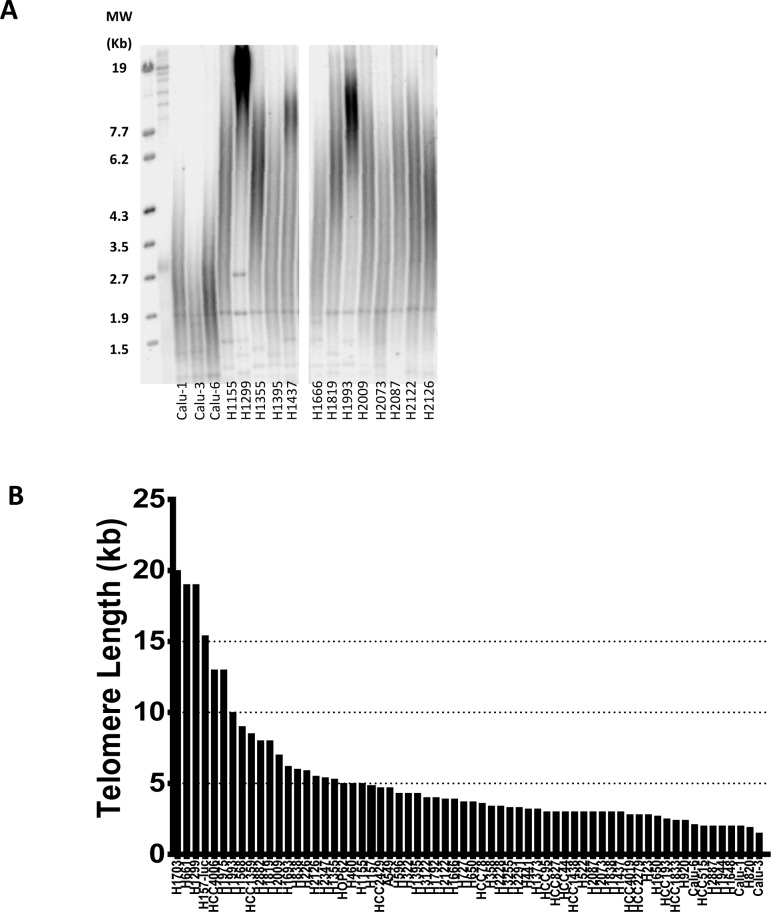
Heterogeneity of telomere length in NSCLC **A.** Representative selection of telomere length variation in NSCLC cell lines *via* Southern blot. **B.** NSCLC panel average telomere length as measured by TRF (values listed in Supplemental Table S1). The names of the cell lines generated at NIH are officially NCI-Hxxxx and here abbreviated as Hxxxx. The lines generated at the University of Texas Southwestern Medical Center Hamon Center are designated HCCxxxx. Cell lines from other sources use the names given elsewhere.

To better understand the telomere landscape of NSCLC, we used a panel of 63 NSCLC cell lines derived from patients with varying histology, stage, race, age, gender and smoking status (from here on referred to as “patient characteristics”) and oncogenotypes (summarized in Supplemental Table S1 along with important, previously unpublished data on telomere length, colony forming efficacy, doubling times, and percent inhibition of colony formation by imetelstat). Average telomere length for each cell line was measured by TRF (telomere restriction fragment) *via* Southern blot and ranged from 1.5 kb to 20 kb (Figure 1). 75% of the lines had average telomeres of 5.5 kb or less and only 6 had average telomeres longer than 10 kb. Thus, telomeres in most of these cancer cell lines are considerably shorter compared to normal (non-cancerous) adult cells (7-15 kb).

Cell lines were divided into quartiles based on telomere length (Supplemental Figure S2A). The first quartile (Q1) average telomere length is 10.8 kb and is significantly longer than the three remaining quartiles of average 4.5 kb, 3.2 kb, and 2.3 kb for quartile 2 (Q2), quartile 3 (Q3), and quartile 4 (Q4), respectively. There was no correlation between telomere length and patient characteristics or oncogenotype. From the longest quartile (Q1) to the shortest quartile (Q4), average smoking pack years is 58, 45, 45 and 46, respectively, and average patient age is 55, 54, 52 and 48 years old. The age range of patients from which the cell lines were derived was 25 to 73 years old, and overall there was no correlation between age of the patient and telomere length of the NSCLC line.

#### Imetelstat inhibits colony forming ability in a telomere length dependent manner across the entire spectrum of NSCLC oncogenotypes

**Figure 2:**
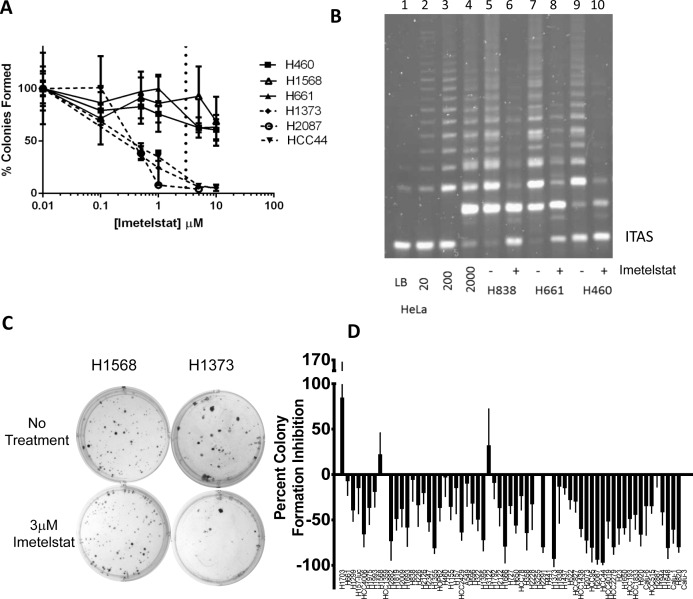
Range of responses to imetelstat treatment in NSCLC colony formation assay screen **A.** Screen dose was determined by dose titrations of 0, 0.1, 0.5, 1, 5, or 10 μM imetelstat in colony formation. 3 μM (the dashed vertical line) was chosen as the dose for the screen. **B.** TRAP assay to verify telomerase inhibition at the conclusion of colony formation assays. Control lanes 1-4, lysis buffer (LB) and 10, 200, 2000, HeLa cells. Lanes 5-6, 2000 H838 cells from colony formation without and with 3 μM imetelstat. Lanes 7-8 and 9-10 show H661 and H460, respectively, without and with imetelstat. ITAS: Internal Telomerase Assay Standard. **C.** Representative colony formation with no treatment *versus* 3 μM imetelstat treatment. **D.** NSCLC cell line colony formation percent inhibition with 3 μM imetelstat. Data represent average of at least 9 replicates; thin line is standard deviation. Values are given in Supplemental Table S1. For comparison, cell lines listed in same order of telomere length (Figure 1B).

The efficacy of imetelstat across the panel of NSCLC cell lines was assessed in a colony formation assay. Preliminary experiments determined 3 μM imetelstat was the optimal screening dose (Figure 2A-2B). In all cases when colonies were harvested and pooled at the end of the colony formation assays, a one-time treatment with 3 μM imetelstat greatly inhibited telomerase activity (Figure 2B). Response of a screen of 63 NSCLC lines ranged from 96% inhibition in colony forming ability to an 84% increase in colony forming ability in the presence of imetelstat (Figure 2C-2D). While the possibility of NSCLC colony growth stimulation by imetelstat was surprising, when the number of untreated colonies was compared to treated colonies for the most resistant NSCLC line, H1703, the difference in colony forming efficiency was not statistically significant (*p* > 0.05). By contrast, for HCC44, the most imetelstat sensitive line, the difference was highly significant (*p* < 0.0001). Thus, the response to imetelstat ranged from 96% inhibition in colony forming ability to no statistically significant response. The response phenotypes did not correlate with patient characteristics or oncogenotype. When response of the NSCLC lines to imetelstat colony formation was compared to telomere length, an overall direct correlation was not observed (Supplemental Figure S2B); however when telomere length quartiles were compared, cell lines with the shortest telomeres (Q4) were more sensitive to imetelstat compared to cell lines with the longest telomeres (Q1) (Supplemental Figure S2C (*p* < 0.03)). Because there was minimal difference in telomere length between Q2, Q3 and Q4, these 3 quartiles were pooled and also show increased response compared to Q1 (Supplemental Figure S2C). While imetelstat response was correlated with colony forming efficiencies of untreated cells and doubling times, the correlation coefficients for these were very modest (r^2^ values of 0.18 and 0.12, respectively, Supplemental Figure S2D-E). The panel of NSCLC lines had legacy data on *in vitro* (MTS assay) response phenotypes for 28 standard and targeted chemotherapies and a range of sensitivity is observed with these drugs. There was no correlation between imetelstat response phenotypes and NSCLC response phenotypes to other therapies (Supplemental Table S2).

This panel of NSCLC cell lines contains “experiments of nature” - two NSCLC cell line pairs derived from the same tumor in each patient before and after neoadjuvant chemotherapy [26]. H1693 (diagnostic mediastinal lymph node biopsy) and H1819 (therapeutic tumor resection after neoadjuvant chemotherapy) were derived 6 months apart [26] and have average telomere lengths of 6.2 and 8 kb, respectively, as well as similar imetelstat colony formation inhibition of ~50%. By contrast, H1993 (diagnostic mediastinal lymph node biopsy) and H2073 (therapeutic tumor resection after neoadjuvant chemotherapy) were derived 3 months apart [26]. They have different telomere lengths of ~10 and 3 kb, for H1993 and H2073, respectively, and different imetelstat colony formation inhibition of 18% and 73%. These two cell line pairs highlight the general trend seen with the panel as a whole that shorter telomeres predict higher imetelstat responsiveness.

#### Long-term imetelstat treatment inhibits telomerase and shortens telomeres in multiple NSCLC cell lines

**Figure 3:**
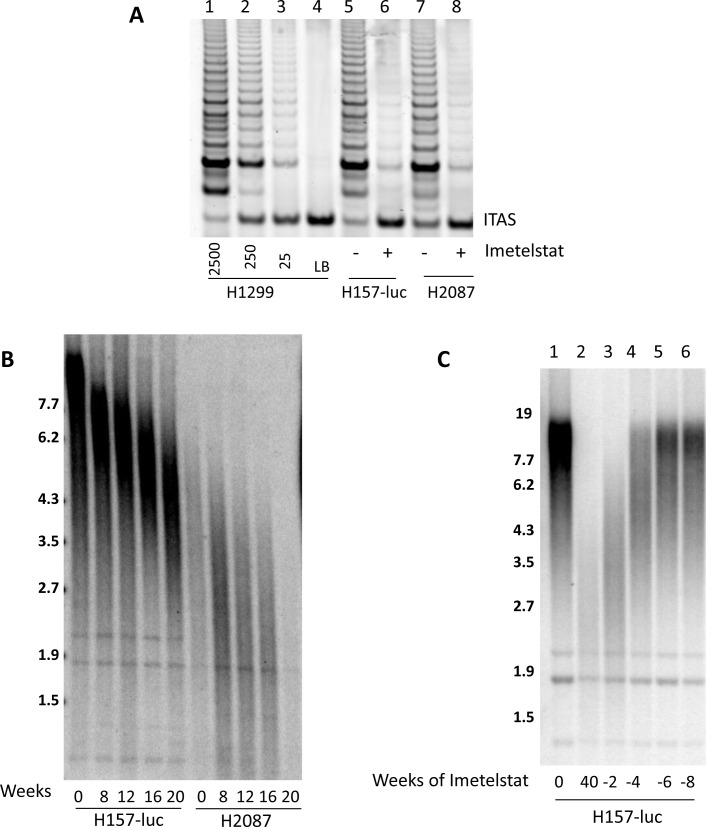
Imetelstat inhibits telomerase and leads to telomere shortening while removal of imetelstat results in regrowth of telomeres NSCLC cell lines H157-luc and H2087 were treated with 1 μM imetelstat. **A.** Telomerase activity after 8 weeks of treatment. Control lanes 1-4, 2500, 250, 25, H1299 cells and lysis buffer. Lanes 5-6, 2500 H157-luc cells and lanes 7-8 are H2087 cells without and with imetelstat for 8 weeks. ITAS: Internal Telomerase Assay Standard. **B.** H157-luc and H2087 parental telomere length and after 8, 12, 16, and 20 weeks of 1 μM imetelstat treatment. **C.** H157-luc telomere length after imetelstat removal. Lane 1 is parental telomere length of H157-luc and lane 2 is H157-luc after 40 weeks of 1 μM imetelstat treatment at which point treatment ceased. Lanes 3-6 are H157-luc 2, 4, 6 and 8 weeks after removal of treatment. Note - H157-luc are NCI-H157 cells with an exogenous luciferase expression construct inserted.

To further explore the correlation of telomere length to imetelstat response, we examined long-term exposure to imetelstat in nine NSCLC cell lines (Calu-3, H157-luc, H358, H460, H1648, H1819, H2087, H2887, and HCC827) which represent a range of telomere lengths, patient characteristics and oncogenotypes (Supplemental Table S1). 1 μM imetelstat was given thrice weekly in mass culture based on the half-life estimates of imetelstat in tissue culture medium. Imetelstat treatment greatly reduced telomerase activity in H157-luc (initial telomere length 15kb) and H2087 (initial telomere length 3 kb) after 8 weeks of continuous treatment (Figure 3A). Telomere length was measured beginning at 8 weeks and then again at 4-week intervals. Irrespective of initial telomere length, telomeres showed progressive shortening by 8 weeks and continued to shorten with prolonged treatment up to 20 weeks, shown in Figure 3B. Similar results were seen in the other cell lines (data not shown).

#### Removal of imetelstat treatment reverses telomere shortening effects

At 40 weeks of treatment, H157-luc cells were still growing in mass culture with no visible changes. To determine if telomere shortening was reversible, imetelstat therapy was stopped after 40 weeks of *in vitro* treatment (~180 population doublings) and cells were grown in the absence of drug. Cells were collected at 2, 4, 6 and 8 weeks after removal of imetelstat and telomere length assessed (Figure 3C). At 40 weeks of imetelstat treatment, telomere length was shortened to 2.6 kb, almost 12 kb shorter compared to the untreated parental cells. After 2 weeks or ~12 population doublings without treatment, telomeres lengthen to 3.4 kb. At 4 weeks (~27 population doublings) after removal of treatment, some H157-luc telomeres returned to parental length and all returned to parental length and remained there after 6 weeks (39 population doublings) in the absence of imetelstat.

#### Long-term imetelstat treatment results in eventual senescence and reduced colony forming ability

**Figure 4:**
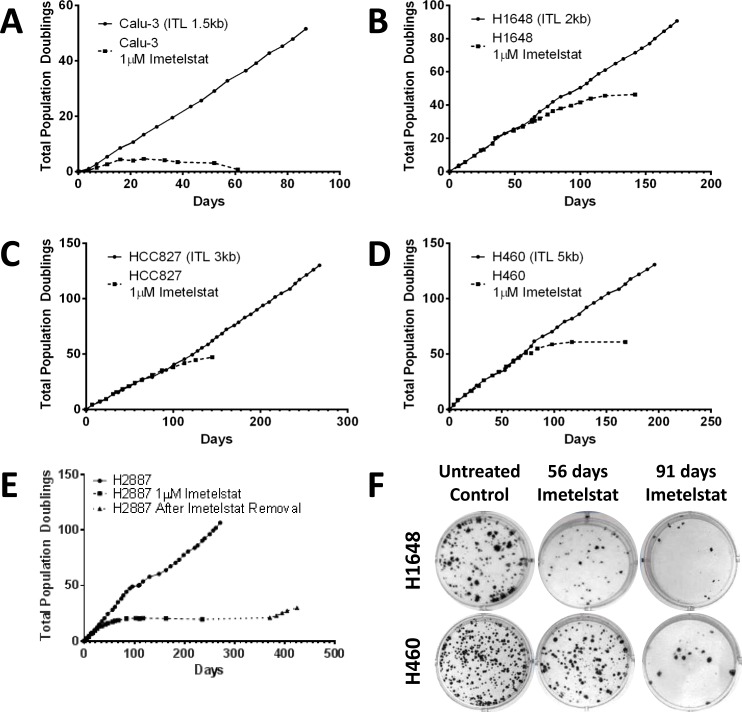
NSCLC growth and colony formation are inhibited after long-term imetelstat treatment Cell lines were grown in the presence and absence of 1 μM imetelstat given thrice weekly. Population doubling growth curves are shown for **A.** Calu-3, initial telomere length (ITL 1.5 kb), **B.** H1648 (ITL 2 kb), **C.** HCC827 (ITL 3 kb), **D.** H460 (ITL 5 kb), and **E.** H2887 (ITL 2 kb). In panel E note H2887 cells were treated long-term with imetelstat and growth slowed in 12 population doublings. At 235 days, imetelstat was removed. Dotted line with triangle indicates growth without imetelstat. **F.** H1648 and H460 liquid colony formation assays for untreated controls and for tumor cells treated with 1 μM imetelstat for 56 and 91 days that demonstrate dramatic loss of colony forming ability after long-term imetelstat treatment.

Continuous *in vitro* treatment of cells in mass culture with imetelstat leads to telomerase inhibition and telomere shortening regardless of initial telomere length, however the necessary treatment time to achieve senescence is dependent on the initial telomere length. Calu-3 had the shortest initial telomeres in the panel (1.5 kb) and the shortest response-time to continuous imetelstat treatment. The growth rate slowed in less than 4 population doublings (11 days) and loss of replicative potential and concomitant decrease in the number of cells followed with continued treatment (Figure 4A). H1648 had the next shortest initial telomere length (2 kb) and responded with slowed growth rate in 15 population doublings at ~2 months of treatment (Figure 4B). H2887, HCC827 and H460 had initial telomere lengths of 2 kb to 5 kb but required ~12 population doublings (~25 days), 40 population doublings (~100 days) and 50 population doublings (~70 days) for growth inhibition, respectively (Figure 4C-4E). An increase in senescence-associated β-galactosidase staining in long-term treated cells compared to parental indicates the slowed growth rate was due to an increase in senescing cells (Supplemental Figure S3).

The effect of shortened telomeres on colony forming ability was tested at multiple time points throughout the long-term treatment. NSCLC H1648 colony forming ability decreased after 56 days of treatment and continued to decrease up to 91 days of treatment (Figure 4F). NSCLC H460, which had longer initial telomeres and required longer imetelstat exposure for cells to senesce, showed minimal difference in colony forming ability after 56 days of treatment. However, by 91 days of treatment, there was a significant reduction in colony forming ability (Figure 4F). In both cell lines, as the telomeres shorten and growth slows, the ability of the cell line to form colonies decreased.

### Long-term imetelstat treatment of NSCLCs has a heterogeneous effect on response to standard chemotherapy and EGFR targeted therapies

Throughout the long-term imetelstat treatment, the cell lines were tested periodically with standard chemotherapies to determine if imetelstat treatment and subsequent telomere shortening resulted in a change in response to other chemotherapies. Cell lines were treated in a 5-day cell viability assay with carboplatin, doxorubicin, gemcitabine, paclitaxel, paclitaxel/carboplatin combination, erlotinib, and vinorelbine - all drugs that have been used in NSCLC treatment. The cell lines initially had a wide range in response to each therapy (Supplemental Table S3). While imetelstat treatment occasionally increased the response to cytotoxic chemotherapy indicated by a change in IC_50_ values, this was highly heterogeneous between tumors and only occasionally gave dramatic decreases in IC50 values (see vinorelbine effect in 3/5 NSCLCs in Supplemental Table S3).

#### Imetelstat slows tumor growth and results in telomere shortening *in vivo* in a telomere length dependent manner

**Figure 5:**
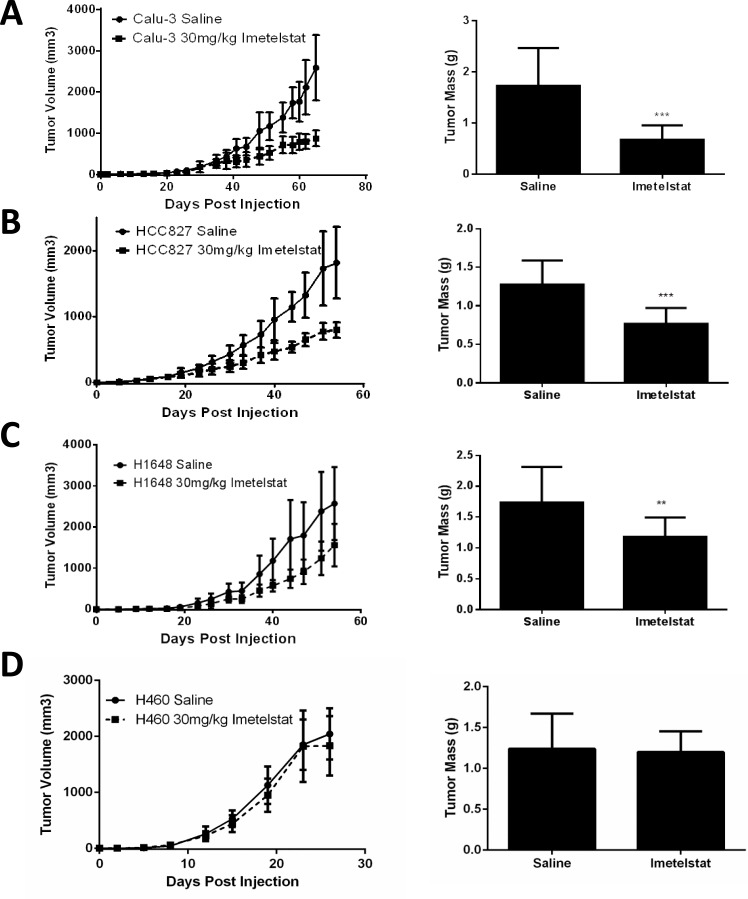
Imetelstat treatment *in vivo* reduces tumor burden **A.** Calu-3 (initial telomere length (ITL) 1.5 kb) tumor growth curves with 30 mg/kg imetelstat *versus* control and final tumor weights (*n* = 10 per group). **B.** HCC827 (ITL 3 kb) tumor growth curves with imetelstat and final tumor weights (*n* = 10 per group). **C.** H1648 (ITL 2 kb) tumor growth curves and final tumor weights (*n* = 12 per group. **D.** H460 (ITL 5 kb) tumor growth curves and final tumor weights. Error bars are standard deviation. ***p < 0.0008, **p < 0.007 as determined by student's *t*-test.

The *in vivo* efficacy of imetelstat was assessed using four cell lines (HCC827, Calu-3, H1648, and H460) in a subcutaneous xenograft model. Calu-3 tumor growth rate was significantly slower in the imetelstat treated group compared to control and final tumor weights were smaller (*p* < 0.0008, Figure 5A). HCC827 and H1648 also showed slower growth rates and decreased tumor size in imetelstat treated tumors *versus* control tumors (*p* < 0.0008 and *p* < 0.007, respectively, Figure 5B-5C).

H460 had the longest telomeres tested *in vivo* and showed no difference in growth before tumors reached maximum tumor burden (Figure 5D), however there was a decrease in telomerase activity and telomere length in the treated tumors (Supplemental Figure S4A-B). H460 is also the fastest growing cell line tested *in vivo* reaching maximum tumor burden in about 3 weeks. To determine if shortened telomeres can reduce tumor forming ability, H460 was pretreated *in vitro* for 12 weeks before tumor cell injection. This pretreatment led to a delay in tumor formation compared to the untreated parental line (Supplemental Figure S4C). Therefore, imetelstat effectiveness *in vivo* is dependent on initial telomere length and treatment duration of imetelstat therapy.

## DISCUSSION

In the present study, we analyzed the efficacy of the telomerase inhibitor imetelstat across a large panel of NSCLC cell lines including differing patient characteristics, oncogenotypes, and telomere lengths. NSCLCs varied ~2 logs in their imetelstat response phenotype in colony formation. Approximately 20% of all NSCLC lines demonstrated dramatic inhibition to imetelstat in the colony formation assay, and this was seen over all NSCLC oncogenotypes. Telomere length ranged from 1.5 kb to 20 kb, but 75% of the panel was less than 5.5 kb and this subset had the greatest response to imetelstat. NSCLC lines treated long-term (up to nine months) include adenocarcinoma, large cell and squamous cell carcinoma histology; Caucasian and African American patients; males and females ranging in age from 25 to 73; a range of oncogenotypes including different combinations of p53, KRAS, LKB1/STK11, and EGFR mutations (Supplemental Table S1), and both sensitive and resistant lines to standard chemotherapy and targeted therapies. Imetelstat effectively inhibited telomerase resulting in shortening of telomeres in all cell lines tested regardless of any clinical or genetic characteristics. This supports inhibition of telomerase as a universal cancer therapy target in telomerase expressing tumors. Time to senescence or cell death, however, was dependent on initial telomere length and growth rate of the cell line. These results were also seen *in vivo* where initial telomere length is proportional to the treatment time necessary to see significant differences in tumor growth rate.

The mechanism of action of imetelstat dictates prolonged treatment is necessary to observe progressive telomere shortening with continued cell division and eventual senescence or cell death. This is accompanied by a lag phase with minimal phenotypic change other than decreased telomere length and suggests shorter telomeres would have faster response times. As expected, initial telomere length correlated with the number of population doublings and time required to achieve critically short telomeres required to trigger these effects both *in vitro* and *in vivo*. Calu-3, H1648, and HCC827 with initial telomere lengths of 1.5 kb, 2 kb, and 3 kb, respectively, all demonstrated slowed growth *in vitro* in a length dependent manner and showed reduced tumor burden in the presence of imetelstat in a xenograft model. H460 (initial telomere length 5 kb) required more population doublings to reach a slowed growth and eventual cell death *in vitro* and showed no initial difference in tumor growth rate in the presence of imetelstat *in vivo*. However, with pre-treatment *in vitro*,** even this originally non-responsive cell line shows a delay in tumor formation emphasizing the need for adequate imetelstat exposure. Taken together, these data support short telomeres as selection criteria for maximum response to imetelstat in patients.

We potentially expected to see resistant sub-populations grow out during the extended culturing *in vitro*; surprisingly we saw no evidence for emerging resistance. To examine resistance in another way, we serial cloned H1648 in the presence of imetelstat, but once again saw no evidence of selection of resistant clones (Supplemental Figure S5). While these experiments are not definitive, they may speak to the unique biology of telomere extension using an RNA template and telomerase. Mutations in either of these required components, which might cause drug resistance, would in fact be predicted to give the same phenotype as the drug itself. Finally, one of the sensitive lines tested herein (H2073) expresses functional multi-drug resistance transporter (MDR) implying that this will not be a mechanism of resistance to imetelstat treatment.

Our results (Figures 3C and 4E) along with Burchett, et al [23] demonstrate the importance of continual treatment. After 40 weeks of treatment, telomeres are significantly reduced in length in H157-luc cells but telomeres regrew in as few as two weeks when imetelstat exposure was stopped. In H2887 cells, imetelstat was removed after 140 days of minimal growth/senescence and post removal of imetelstat, the cell line eventually recovers. Thus, once imetelstat treatment is begun and telomerase is inhibited, cells must be exposed to uninterrupted telomerase inhibition to ensure not only continued telomere shortening but also prevention of telomere re-elongation.

Although this study shows that all cell lines tested with imetelstat experienced a decrease in telomere length and concomitant loss of replicative potential, this study also indicates that given the therapeutic window, tumors with short telomeres will respond best to imetelstat therapy (Figure 5, Supplemental Figure S4). Indeed, the clinical trial of imetelstat in NSCLC provided information on a trend in which patients with the shortest telomeres responded best to therapy [25]. This study used imetelstat as maintenance therapy following standard chemotherapy. Due to the lag time necessary to achieve tumor cell death, imetelstat may work best in combination with first line therapy and continued as a maintenance therapy with other cytotoxic or targeted chemotherapies. In some cases long-term imetelstat can sensitize NSCLCs to cytotoxic therapies (Supplemental Table S3). Previous work has shown imetelstat can sensitize breast cancer to radiation [16, 27] or trastuzumab [28] and breast and liver cancer to doxorubicin [16, 19]. While further studies will be required to determine the optimal combinations that are clinically relevant, some of our data supports the idea that imetelstat might best be used in combination with EGFR targeted therapy. For example, tumors with EGFR mutations making them susceptible to EGFR tyrosine kinase inhibitors (EGFR TKIs) have shown great initial response to EGFR TKIs in the clinic but frequently develop resistance [29]. HCC827, a NSCLC EGFR mutant line responsive to erlotinib [30], was effectively targeted with imetelstat and responded in a telomere length dependent manner both *in vitro* and *in vivo*. Imetelstat in combination with or following erlotinib treatment in EGFR mutant cancers would be predicted to work concomitantly by treating the EGFR mutant cancer cells with erlotinib while concurrently shortening telomeres. Cells that eventually become resistant to EGFR therapy would thus have short telomeres preventing their continued growth. Therefore, follow up studies should include combining imetelstat with targeted therapies such as erlotinib in EGFR mutant lung cancer and crizotinib in EML4-ALK fusion lung cancer. Additionally, with the advances of immunotherapeutic approaches to lung cancer that have recently developed, it will be important to determine if targeting telomerase also changes the immune response to lung cancer cells after various immunotherapies.

Our results, along with previous imetelstat studies, support continued investigation of telomerase inhibition in NSCLC, particularly for tumors with short telomeres. This could be with imetelstat or other telomerase targeting agents. Based on the NSCLC panel we studied, the subset of NSCLC potentially sensitive to telomerase-targeted therapy could be the large majority of all NSCLC patients. We emphasize that imetelstat and thus telomerase-targeted therapy can be effective in NSCLCs with many different oncogenotypes and drug response phenotypes. In addition, imetelstat dosage schedules must ensure continuous treatment so drug induced telomere shortening is not negated by drug free intervals allowing telomere regrowth. In conclusion, the present results support continued testing of telomerase targeted therapy across the full spectrum of NSCLCs, with any early phase clinical trials directed at patients shown to have the shortest quartile of telomere length at the initiation of therapy to provide an enrollment enrichment biomarker for telomerase targeted cancer therapy.

## MATERIALS AND METHODS

### Tumor cell lines and cell culture

With the exception of Calu-1, Calu-3, Calu-6, A549 and HOP62 (obtained from ATCC), all other cell lines were established in the lab of Drs. John Minna and Adi Gazdar at the NIH (referred to as NCI-Hxxxx, or Hxxxx) or UT Southwestern Medical Center (referred to as HCCxxxx) (1981-2014) and are available through ATCC or the Hamon Center. Cells were cultured in RPMI with 5% fetal bovine serum in 5% CO_2_ at 37°C. H157-luc expresses firefly luciferase which was established as previously described [24] and was used in some experiments because its telomere length is 15.4 kb compared to H157 parental line whose telomere length is 4.8 kb. All cell lines were authenticated by DNA fingerprinting with the Promega StemElite ID system (Cat# G9530) and confirmed mycoplasma free by e-Myco kit (Boca Scientific). Imetelstat (Geron Corp) treatment began 24 hours after seeding and fresh drug (1μM) in PBS was given 2-3 times per week for continuous treatment. Population doubling (PD) was calculated as [(log N(t) -logN(t0))/log2] where N(t) is the number of cells at time of passage and N(t0) is the number of cells seeded at previous passage. Doubling times were calculated by dividing number of hours from seeding to harvest by the PD.

### Telomerase activity

Telomerase activity was measured using the telomerase repeat amplification protocol (TRAP) assay *via* the TRAPeze Telomerase Detection Kit (Millipore). 100,000 cells were resuspended in 100 μl 1x CHAPS lysis buffer. 1 μl (1000 cells) was used for the PCR reaction. For tumor samples, 5 μg of protein were used.

### Telomere length

Telomere length was determined using a Southern blot protocol as previously described [31]. Briefly, DNA was obtained using the DNeasy kit (Qiagen) in the QiaCube (Qiagen). 1 μg DNA was digested with a mix of equal parts AluI (New England Biosciences), CfoI (Promega), HaeIII (New England Biosciences), HinfI (New England Biosciences), MspI (New England Biosciences), and RsaI (New England Biosciences) restriction enzymes. The digested DNA was run on 0.7% agarose gel. The gel was denatured, dried, neutralized and hybridized with a radioactive probe to the telomeric sequence, exposed to a phosphor screen and scanned with a phosphor Imager.

### Colony formation

500 cells were plated in triplicate in 6-well plates. The assay was terminated at 14-21 days when at least 50% of colonies had 50 or more cells per colony in control wells. Cells were fixed and stained with 6% glutaraldehyde and 0.5% crystal violet solution [32]. Colonies were counted by eye and confirmed by imaging plates with the ChemiDoc XRS+ Imager (Bio-Rad) and using the Colony Counting function of Quantity One Software (v4.6.5, Bio-Rad). Each assay was repeated a minimum of 3 times per cell line. For the panel screen, 3 μM imetelstat was added 24 hours after seeding. Long-term treated cells did not receive imetelstat in the assay. Percent colony forming efficiency was calculated as (number of colonies counted/number of cells seeded)*100.

### SA-β-gal staining

Senescence associated β-galactosidase staining was performed as previously described [33].

### Tumor xenografts

Mouse work followed an IACUC approved protocol. 1×10^6^ cells were injected subcutaneously into the right flank of 6-8 week old female NOD/SCID mice. Mice were randomized with 10 mice per group and treatment began 1 day after cell injection. 30 mg/kg imetelstat in saline or saline alone was given in 100 μL i.p. 3x/week [17, 34, 35]. Tumors were measured using calipers to take perpendicular length measurements 2x/week. Tumor volumes were calculated as (L x w2) (π/6) where L is length and w is width, the shorter measurement. Mice were sacrificed when control tumors reached an average of 2000 mm^3^ when tumors were harvested and weighed. For the pre-treatment studies, H460 cells were treated with 1 μM imetelstat for 12 weeks *in vitro* before injection and imetelstat treatment was not continued *in vivo*.

## SUPPLEMENTARY MATERIALS FIGURES AND TABLES


