# Supplementary material for: Telomerase inhibitor imetelstat has preclinical activity across the spectrum of non-small cell lung cancer oncogenotypes in a telomere length dependent manner
Source: Oncotarget. 2016 May 12;7(22):31639–51. doi: 10.18632/oncotarget.9335 (PMC5077965; doi:10.18632/oncotarget.9335)
Supplement: Supplementary file 1 [file oncotarget-07-31639-s001.pdf]

Telomerase inhibitor imetelstat has preclinical activity across the spectrum of non-small cell lung cancer oncogenotypes in a telomere length dependent manner

Supplementary Material

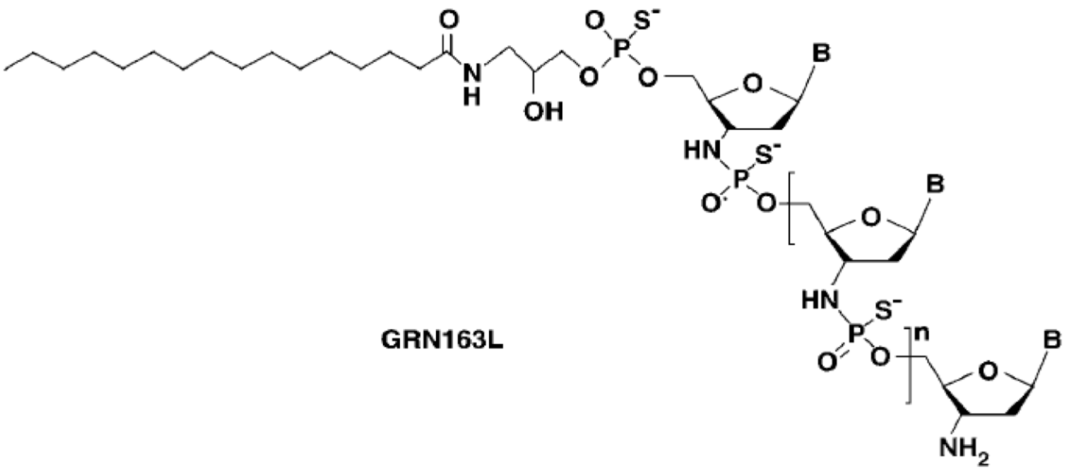

Supplemental Figure S1. Structure of Imetelstat

First published in Herbert BS, et al. Lipid modification of GRN163, an N3'-->P5' thio-phosphoramidate oligonucleotide, enhances the potency of telomerase inhibition. Oncogene. 2005;24:5262-8.

Supplemental Table S1.

CFI – Colony Formation Inhibition, CFE – Colony Forming Efficiency, SD – Standard Deviation, DT – Doubling Time, ADN – adenocarcinoma, HD - Homozygous Deletion, MEC - Muco-epidermoid carcinoma, LCN – large cell neuroendocrine, ADSQ – adenosquamous, SCC- squamous cell carcinoma, LCC – large cell, M – mesothelioma, CE - Carcinoid-endocrine, C – Caucasian, PY – smoking pack year, B – Black, A - Asian, NC –No Colonies Formed, LC – Low Colony (colony formation efficiency was <1%); Blank cells indicate we do not have the data.

Cell Line	Tumor Type	Stage	Age	Race	Gender	Smoking (PY)	p53	KRAS	STK11	EGFR	Telo Length (kb)	% CFI	SD	% CFE	SD	DT (hr)	SD
**A549**	ADN		58	C	M		WT	Mut	Mut	WT	4.7	9	27	81	23	24	3
**Calu-1**	MEC		47	C	M		HD	Mut	WT	WT	2	60	17	27	3	50	15
**Calu-3**	ADN		25	C	M		Mut	WT	WT	WT	1.5			LC		40	6
**Calu-6**	ADN		61	C	F		Mut	Mut	WT	WT	2.1	34	25	6	2	32	5
**H23**	ADN		51	B	M	40PY	Mut	Mut	Mut	WT	2.8	59	11	32	6	39	4
**H157**	SCC	3B	59	C	M	Y	Mut	Mut	Mut	WT	4.8	30	26	47	14	21	2
**H157-luc**	SCC	3B	59	C	M	Y	Mut	Mut	Mut	WT	15.4	14	27				
**H226**	S M				M		Mut	WT	WT	WT	5.9	33	23	41	17	52	13
**H322**	ADN	4	52	C	M	60PY	Mut	WT	WT	WT	4.3	49	12	60	12	51	7
**H358**	ADN			C	M		HD	Mut	WT	WT	3.4	64	17	36	12	38	10
**H441**	ADN	3A	33		M		Mut	Mut	WT	WT	3.2			LC		47	8
**H460**	LCC				M		WT	Mut	Mut	WT	5	3	23	57	23	21	3
**H522**	ADN	2	60	C	M	60PY	Mut	WT	WT	WT	3	28	8	42	2	48	4
**H596**	ADSQ	3A	73	C	M		Mut	WT	WT	WT	4.3	31	24	36	11	41	8
**H650**	ADN				M	N	Mut	Mut	WT	WT	3.7	56	4	12	2	49	3
**H661**	LCC	3B	43	C	M		Mut	WT	WT	WT	19	7	18	43	7	33	8
**H727**	CE	3A	65	C	F	60PY	WT	Mut	Mut	WT	3.7	34	5	74	15	41	3
**H820**	ADN	4	53	C	M		Mut	WT	WT	Mut	1.9	79	4	13	6	65	28
**H838**	ADN	3B	59	C	M	80PY	Mut	WT	Mut	WT	6	5	25	82	11	26	2
**H920**	ADN	4	44	C	M	75PY	Mut	WT	WT	WT	2.4	66	14	57	14	42	7
**H1155**	LCN	3A	36	C	M	20PY	Mut	Mut	WT	WT	5	35	13	80	19	23	2
**H1299**	LCN	3A	43	C	M	50PY	HD	WT	WT	WT	19	39	8	26	7	23	3
**H1355**	ADN	4	53	C	M	100PY	Mut	Mut	Mut	WT	5.3	81	2	66	15	49	21
**H1373**	ADN	3A	56	B	M	30PY	Mut	Mut	WT	WT	3.2	92	8	10	3	37	7
**H1395**	ADN	2	55	C	F	15PY	WT	WT	Mut	WT	4.3	72	13	14	3	51	9
**H1437**	ADN	1	60	C	M	70PY	Mut	WT	Mut	WT	3	14	6	94	10	29	4
**H1568**	ADN	4	48	C	F	60PY	Mut	WT	HD	WT	9	-22	22	23	3	43	10
**H1648**	ADN	3A	39	B	M	40PY	Mut	WT	WT	WT	2	80	12	6	4	42	10
**H1650**	ADN	3B	27	C	M	10PY	Mut	WT	WT	Mut	2.7	58	6	26	2	40	4
**H1666**	ADN	3	50	C	F		WT	WT	WT	WT	3.9	79	20	5	2	45	11
**H1693**	ADN	3B	55	C	F	80PY	Mut	WT	WT	WT	6.2	58	23	22	4	51	18
**H1703**	ADSQ	1	56	C	M	50PY	Mut	WT	WT	WT	20	-84	90	17	11	48	14
**H1792**	ADN	4	50	C	M	30PY	Mut	Mut	WT	WT	4	9	16	61	8	33	3
**H1819**	ADN	3	55	C	F	80PY	Mut	WT	WT	WT	8	48	6	35	22	51	13
**H1838**	ADN				F		Mut	WT	WT	WT	3	13	33	2	1	72	9
**H1944**	ADN	3B	62	C	F	40PY	WT	Mut	Mut	WT	2	41	11	76	13	38	7
**H1975**	ADN				F	N	WT	WT	WT	Mut	13	36	5	32	5	42	8
**H1993**	ADN	3A	47	C	F	30PY	Mut	WT	Mut	WT	10	18	8	72	12	32	6
**H2009**	ADN	4	68	C	F	30PY	Mut	Mut	WT	WT	7	37	22	35	8	30	8
**H2073**	ADN	3A	47	C	F	30PY	Mut	WT	Mut	WT	3	72	14	42	6	45	7
**H2087**	ADN	1	69	C	M	60PY	Mut	WT	WT	WT	3	93	5	32	16	51	5
**H2122**	ADN	4	46	C	F	30PY	Mut	Mut	Mut	WT	3.9	36	20	44	10	32	8
**H2126**	ADN	3B	65	C	M		Mut	WT	Mut	WT	5.5	20	9	38	6	41	10
**H2228**	ADN				F	N	Mut	WT	WT	WT	3.4	32	29	62	13		
**H2291**	ADN				M	N	Mut	Mut	WT	WT	3.3	79	5	23	7		
**H2347**	ADN	1	54	C	F		WT	WT	WT	WT	5.4	52	5	38	10	39	7
**H2882**	NSCLC	4	61		F		Mut	WT	WT	WT	8	73	25	19	10	35	6
**H2887**	NSCLC	4	31		M		Mut	Mut	WT	WT	2	0	7	77	7	43	8
**H3122**	NSCLC				M		Mut	WT	WT	WT	4	-31	31	7	3	48	9
**H3255**	ADN	3B	47	C	F	N	Mut	WT	WT	Mut	3.3			NC			
**HCC44**	ADN		54	C	F		Mut	Mut	Mut	WT	3	96	3	7	1	37	3
**HCC78**	ADN		55	C	M		Mut	WT	WT	WT	3.6	23	14	35	10	39	7
**HCC95**	SCC	4	65	C	M		Mut	WT	WT	WT	3	80	10	12	5	41	3
**HCC193**	ADN	4	71	C	F		Mut	WT	WT	WT	2.5	41	23	7	3	45	13
**HCC515**	ADN	3B	39	C	F		Mut	Mut	Mut	WT	2	34	4	49	8	39	3
**HCC827**	ADN		38	C	F		Mut	WT	WT	Mut	3	29	6	16	8	44	13
**HCC1359**	LCC	4	55	B	F	37PY	Mut	WT	WT	WT	8.5			NC		63	2
**HCC1438**	LCC	1A	43	B	M	N	WT	WT	WT	WT	3	59	4	19	4	37	4
**HCC1833**	ADN	1B	69	C	F	30PY	WT	WT	Mut	WT	2.4	44	18	76	23	47	3
**HCC2279**	ADN	2B	52	A	F	40PY	Mut	WT	WT	Mut	2.8	79	6	9	2	49	8
**HCC2429**	NSCLC				F		Mut	WT	WT	WT	4.7	63	8	116	15		
**HCC4006**	ADN			C	M	N	Mut	WT	WT	Mut	13	65	15	23	2	47	5
**HCC4019**	ADN	4	40	C	M	50PY	Mut	Mut	WT	WT	2.8	51	20	21	3	61	7
**HOP62**	ADN				F		Mut	Mut		WT	5	36	12	15	3	30	7

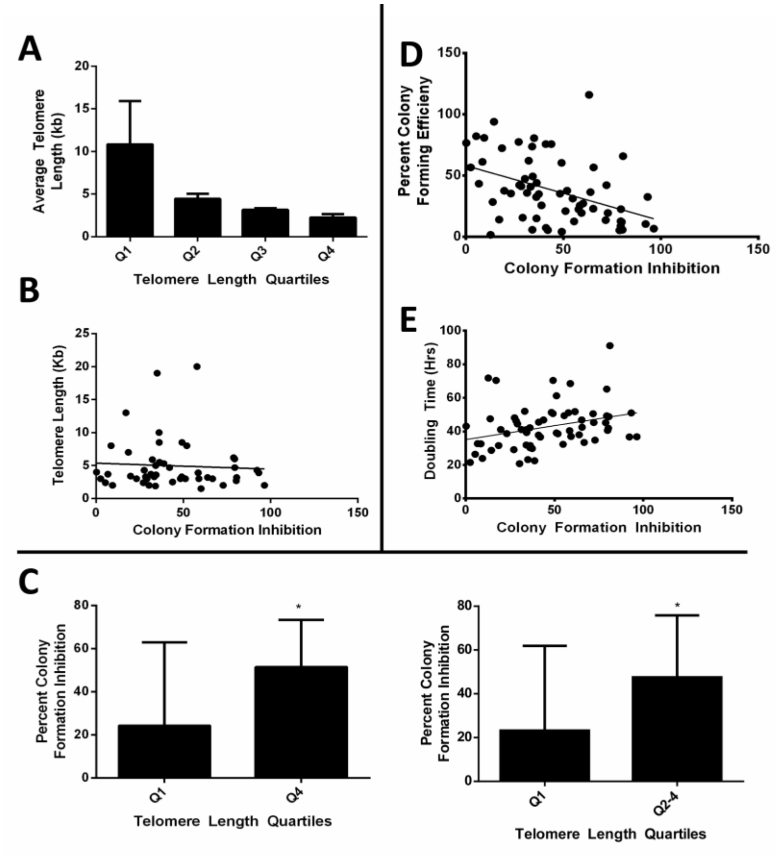

Supplemental Figure S2. Telomere Length Quartiles and Colony Formation Inhibition Correlations

(A) NSCLC panel divided into quartiles based on telomere length. Average telomere length of each quartile: Q1 – 10.8 kb, Q2 – 4.5 kb, Q3 – 3.2 kb, Q4 – 2.3 kb. (B) Correlation between telomere length and colony formation inhibition of the panel (r^2 = 0.003) (C) Q4 (shortest telomeres) is more sensitive to imetelstat in colony formation than Q1 (longest telomeres) ($p>0.03$) and combination of Q2, Q3, and Q4 is more sensitive to imetelstat colony formation than Q1 ($p>0.01$). (D) Correlation between colony forming efficiency and colony formation inhibition of the panel (r^2 = 0.18) (E) Correlation between doubling time of the cell line and colony formation inhibition of the panel (r^2 = 0.12).

Supplemental Table S2. Correlation of Imetelstat Colony Formation Inhibition to IC50s of Other Therapies

IC50s for each cell line from 26 different therapies were correlated to imetelstat colony formation reduction with imetelstat. The r^2 value is listed for each therapy comparing IC50s versus imetelstat colony formation inhibition across the panel.

Drug	r^2 value
AZD6244	0.020
Carboplatin	0.018
Cetuximab	0.011
Chloroquine	0.002
Cisplatin	0.016
Cyclopamine	0.025
Docetaxel	0.018
Doxorubicin	0.014
Etoposide	0.023
Gefitinib	0.098
Gemcitabine	0.004
Gemcitabine/Cisplatin	0.016
Irinotecan	0.077
Lapatinib	0.148
MK-2206	0.000
Nintedanib	0.001
Olaparib	0.000
Paclitaxel	0.006
Paclitaxel/Carboplatin	0.001
PD173074	0.170
Pemetrexed	0.006
Pemetrexed/Cisplatin	0.010
PF2341066	0.078
Rapamycin	0.003
Smac Mimetic	0.016
Sorafenib	0.043
Erlotinib	0.134
Vinorelbine	0.018

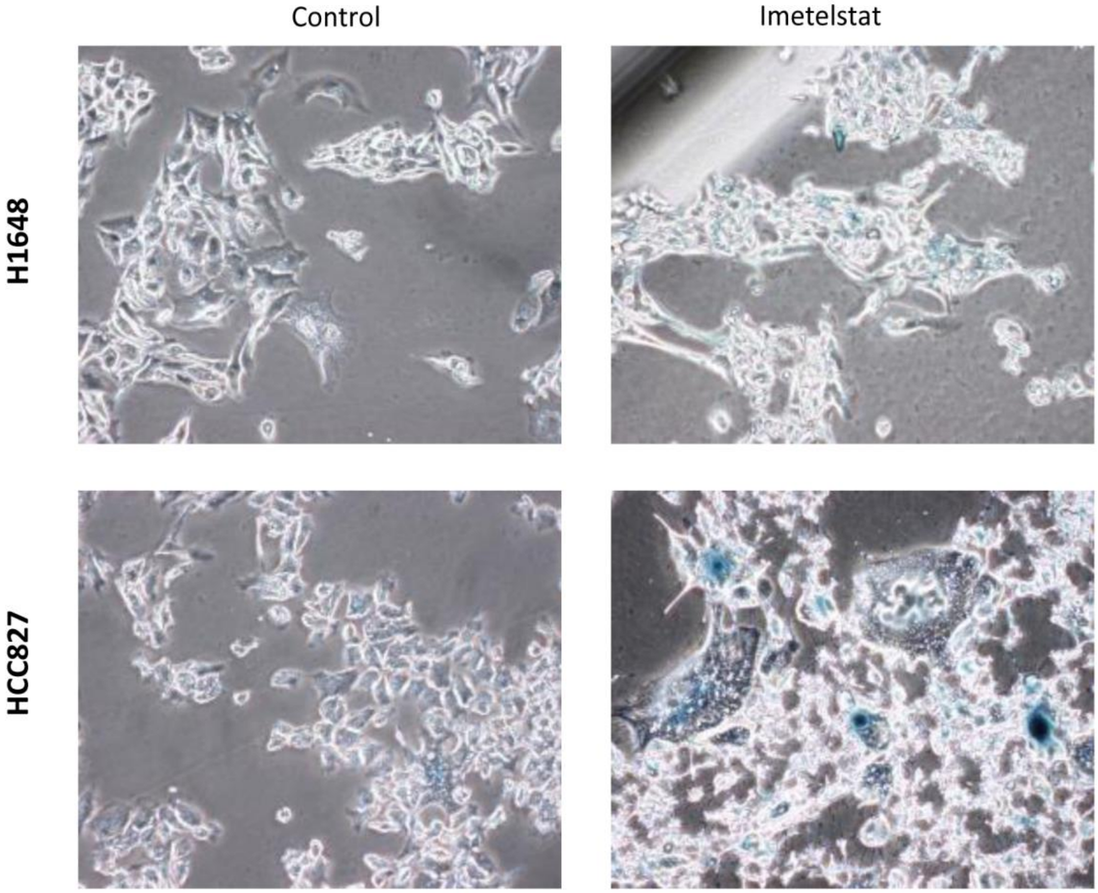

Supplemental Figure S3. Senescence Associated β-galactosidase Staining of Long-term Imetelstat Treated Cells

Senescence associated β-galactosidase staining for control and imetelstat treated H1648 cells at 110 days of treatment and HCC827 cells at 130 days of treatment.

Supplemental Table S3.

NSCLCs were treated long-term with 1 μM imetelstat and drug response phenotypes to a variety of chemotherapy and targeted therapy agents were determined using a 96-well MTS cell viability assay. Drugs were tested at 8 different drug concentrations each in octuplicate and assays were repeated three times. IC50s are displayedin the tablebelow. Imetelstat treatment occasionally increases the response to cytotoxic chemotherapy, but this is highly heterogeneous between tumors.

[illegible]

Cell Line	Carboplatin (μM)	Doxorubicin (nM)	Gemcitabine (nM)	Paclitaxel (nM)	Paclitaxel+ Carboplatin (nM)	Erlotinib (μM)	Vinorelbine (nM)
H157 0wks	45	26.5	3.6	0.41	0.385	24.5	0.235
H157 8wks	36.5	23	3.04	0.37	1.3	16	0.16
H157 16wks	56.5	27.5	5.25	0.22	0.185	13.5	0.025
H1648 0wks	49.5	255	27	5.25	4.25	10	1.95
H1648 6wks	40.5	145	22	1.3	1.9	3.05	0.018
H1819 0wks	170	92.5	1.4	1.95	5.4	0.375	0.036
H1819 8wks	125	220	2.4	1.36	1.98	4.85	0.014
H1819 16wks	120	160	7.55	0.45	0.27	1.12	0.037
H2087 0wks	5.05	37	2.75	0.37	0.29	2.3	0.125
H2087 16wks	4.8	40	2.95	0.37	0.27	7.1	0.435
H358 0wks	33.5	100	3.2	1.2	3.7	3.55	5.9
H358 6wks	15.5	8.5	1.5	0.635	0.985	0.66	0.032

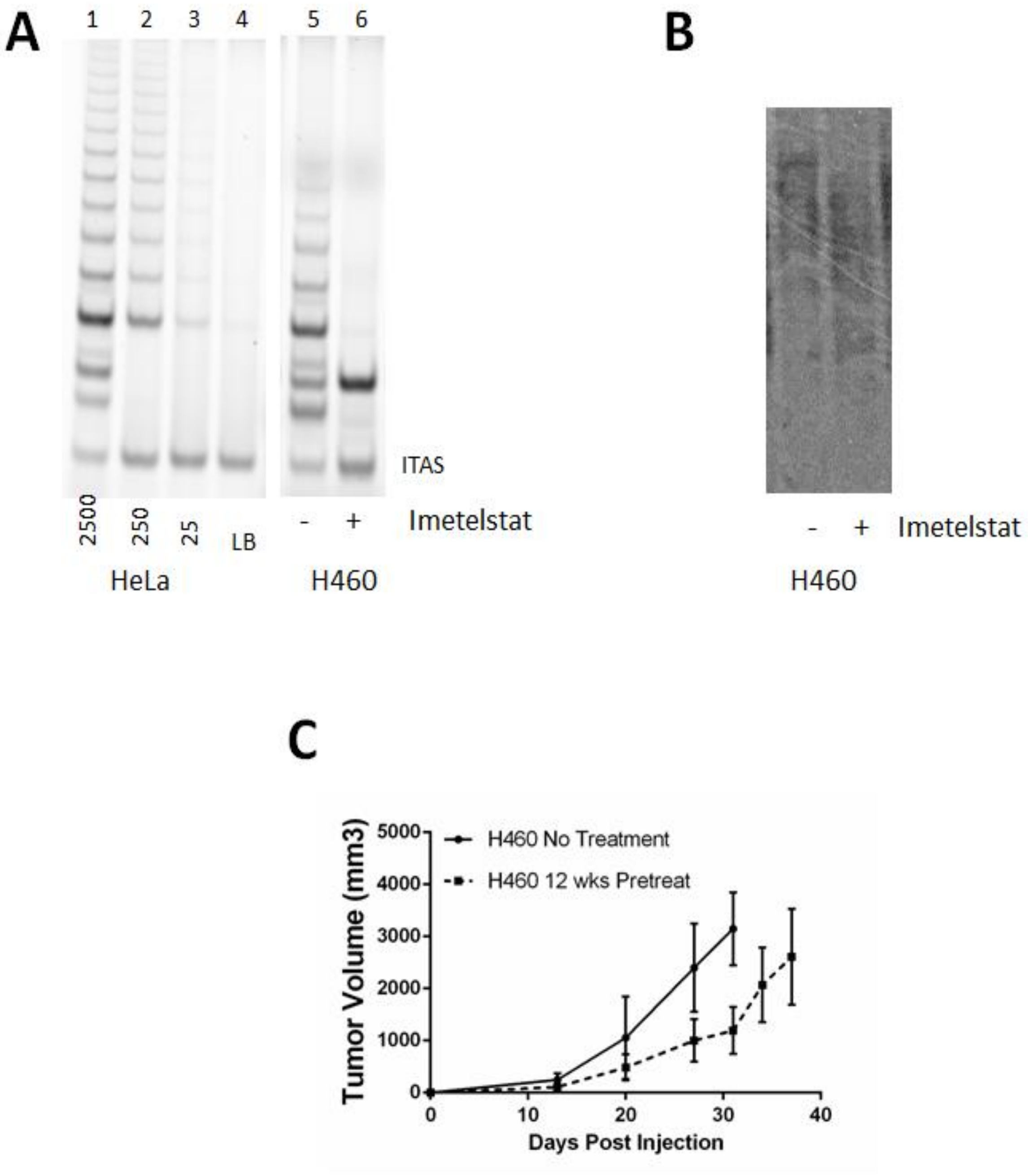

Supplemental Figure S4. Imetelstat Therapy in vivo Response is Dependent on Initial Telomere Length and Therapy Duration.

(A) TRAP assay comparing telomerase activity of H460 tumors without (lane 5) and with (lane 6) imetelstat treatment. Control lanes 1-4, 2500, 250, 25 HeLa cells and lysis buffer

(LB). ITAS, Internal Telomerase Assay Standard. (B) TRF of tumors from mice receiving saline (left column) or 30 mg/kg imetelstat (right column) showing a decrease in telomere length with imetelstat treatment. (C) Xenograft tumor growth curves of parental H460 or H460 cells pretreated with imetelstat for 12 weeks *in vitro* before tumor cell injection. No treatment was given during xenograft growth.

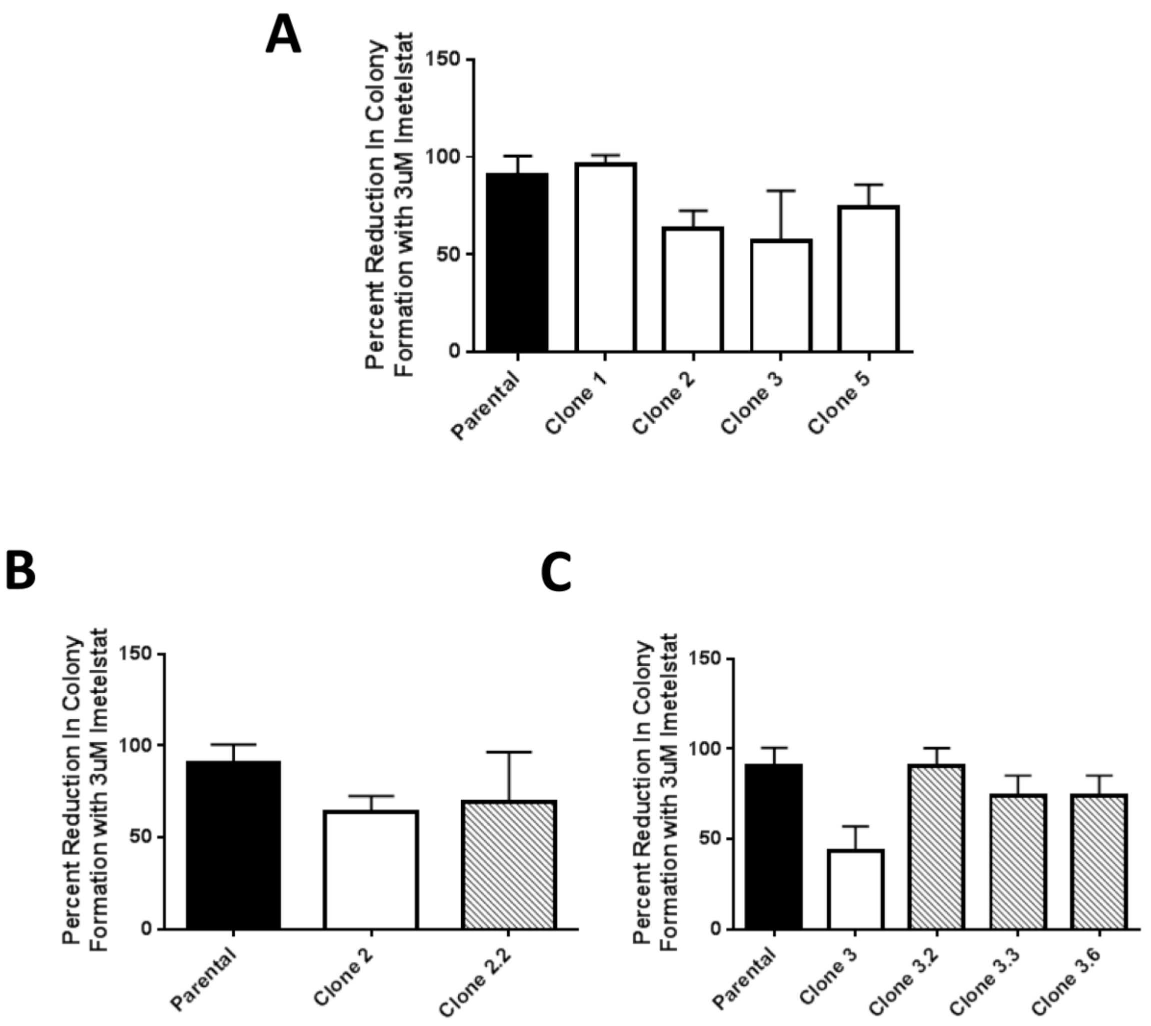

Supplemental Figure S5. Serial Cloning of H1648 in the Presence of Imetelstat. (A) H1648 reduction in colony formation of parental line (black) or clones of H1648 (white) selected in the presence of 3 μM imetelstat. (B) Percent reduction in colony forming ability of parental (black), Clone 2 (white) and Clone 2.2 (striped), a subclone of Clone 2. (C) Percent reduction in colony forming ability of H1648 parental (black), Clone 3 (white) and Clone 3 subclones 3.2, 3.3, and 3.6 (striped).

Supplemental Methods

5-day Cell Viability Assay

2000 cells per well were plated in 96-well plate format. Drugs were added in 4-fold dilutions with a maximum dose of 1000 nM for paclitaxel (Bristol-Myers Squibb) and vinorelbine (Pierre Fabre Company), 2000 nM for doxorubicin (Teva Parenteral) and gemcitabine (Eli Lilly and Company), 42.5 μM for imetelstat (Geron Corp), 808 μM for carboplatin (Teva Parenteral), and 100 μM for cisplatin (Teva Parenteral) and erlotinib (OSI Pharmaceuticals).
Paclitaxel/carboplatin combination was given in a 2:3 wt/wt ratio. Each drug was given at 8 drug concentrations per assay. Plates were incubated for four days and relative cell number was determined by incubating for 1 to 3 hours at 37°C in the presence of MTS (Promega, Madison, WI), final concentration 333 μg/ml. Absorbance readings of the plate were obtained at 490 nm using a Spectra Max 190 (Molecular Devices). Each plate contained eight replicates per concentration and was repeated at least 4 times. Drug sensitivity curves and IC50s were calculated using in-house software, DIVISA.

Serial Cloning

1000 H1648 cells were plated in a 10cm dish in the presence of 3 μM imetelstat. 8 mm cloning cylinders lined with silicone grease were placed over surviving clones. 10 μL trypin were added to the center of the ring and 50 μL of RPMI +5% FBS media was used to harvest the cells and transfer to a new dish. These cells were allowed to proliferate and then used in the standard colony formation already described in the materials and methods. The colony formation assay was repeated a minimum of two times with each replicate done in triplicate. Subclones of these clones were then performed using this same protocol.
